# Comparison of Cytomegalovirus-Specific Immune Cell Response to Proteins versus Peptides Using an IFN-γ ELISpot Assay after Hematopoietic Stem Cell Transplantation

**DOI:** 10.3390/diagnostics11020312

**Published:** 2021-02-15

**Authors:** Eva Wagner-Drouet, Daniel Teschner, Christine Wolschke, Kerstin Schäfer-Eckart, Johannes Gärtner, Stephan Mielke, Martin Schreder, Guido Kobbe, Inken Hilgendorf, Stefan Klein, Mareike Verbeek, Markus Ditschkowski, Martina Koch, Monika Lindemann, Traudel Schmidt, Anne Rascle, Sascha Barabas, Ludwig Deml, Ralf Wagner, Daniel Wolff

**Affiliations:** 1 Department of Hematology, Medical Oncology, and Pneumology, University Medical Center of the Johannes Gutenberg University, 55131 Mainz, Germany; eva.wagner@unimedizin-mainz.de (E.W.-D.); daniel.teschner@unimedizin-mainz.de (D.T.); 2 Department of Stem Cell Transplantation, University Medical Center Hamburg-Eppendorf, 20246 Eppendorf, Hamburg, Germany; wolschke@uke.de; 3 Medizinische Klinik 5, Klinikum Nürnberg Nord, Paracelsus Medizinische Privatuniversität, 90419 Nürnberg, Germany; kerstin.schaefer-eckart@klinikum-nuernberg.de (K.S.-E.); johannes.gaertner@klinikum-nuernberg.de (J.G.); 4 Department of Medicine II, University Medical Center Würzburg, 97080 Würzburg, Germany; stephan.mielke@ki.se (S.M.); martin.schreder@wienkav.at (M.S.); 5 Department of Laboratory Medicine, CAST, Karolinska Institutet and University Hospital, 17177 Stockholm, Sweden; 6 Department of Hematology, University Hospital Düsseldorf, Medical Faculty, Heinrich Heine University, 40225 Düsseldorf, Germany; Kobbe@med.uni-duesseldorf.de; 7 Klinik für Innere Medizin II, Abteilung für Hämatologie und Internistische Onkologie, Universitätsklinikum Jena, 07747 Jena, Germany; Inken.Hilgendorf@med.uni-jena.de; 8 Department of Hematology and Oncology, UMM University Medical Center Mannheim, University of Heidelberg, 68167 Mannheim, Germany; stefan.klein@umm.de; 9 Medical Department, Hematology and Oncology, Klinikum rechts der Isar, Technical University Munich, 81675 Munich, Germany; mareike.verbeek@mri.tum.de; 10 Innere Klinik, Tumorforschung, University Hospital Essen, 45147 Essen, Germany; markus.ditschkowski@uk-essen.de; 11 Department of Hepatobiliary Surgery and Transplantation, University Medical Center Hamburg-Eppendorf, 20246 Eppendorf, Hamburg, Germany; martina.Koch@unimedizin-mainz.de; 12 Institute for Transfusion Medicine, University Hospital Essen, 45147 Essen, Germany; monika.lindemann@uk-essen.de; 13 Lophius Biosciences, 93053 Regensburg, Germany; traudel.schmidt@lophius.com (T.S.); anne.rascle@lophius.com (A.R.); sascha.barabas@lophius.com (S.B.); ludwig.deml@lophius.com (L.D.); 14 Institute of Clinical Microbiology and Hygiene, University Medical Center Regensburg, 93053 Regensburg, Germany; 15 Department of Internal Medicine III, Hematology and Oncology, University Medical Center Regensburg, 93053 Regensburg, Germany

**Keywords:** CMV, CMV-specific cellular immunity, hematopoietic stem cell transplantation, recall antigen, peptide, immune monitoring, IFN-γ ELISpot, T cells, antigen processing and presentation, immunosuppression

## Abstract

Cytomegalovirus (CMV) infection is a major cause of morbidity and mortality following hematopoietic stem cell transplantation (HSCT). Measuring CMV-specific cellular immunity may improve the risk stratification and management of patients. IFN-γ ELISpot assays, based on the stimulation of peripheral blood mononuclear cells with CMV pp65 and IE-1 proteins or peptides, have been validated in clinical settings. However, it remains unclear to which extend the T-cell response to synthetic peptides reflect that mediated by full-length proteins processed by antigen-presenting cells. We compared the stimulating ability of pp65 and IE-1 proteins and corresponding overlapping peptides in 16 HSCT recipients using a standardized IFN-γ ELISpot assay. Paired qualitative test results showed an overall 74.4% concordance. Discordant results were mainly due to low-response tests, with one exception. One patient with early CMV reactivation and graft-versus-host disease, sustained CMV DNAemia and high CD8^+^ counts showed successive negative protein-based ELISpot results but a high and sustained response to IE-1 peptides. Our results suggest that the response to exogenous proteins, which involves their uptake and processing by antigen-presenting cells, more closely reflects the physiological response to CMV infection, while the response to exogenous peptides may lead to artificial in vitro T-cell responses, especially in strongly immunosuppressed patients.

## 1. Introduction

Cytomegalovirus (CMV) infection is a serious cause of morbidity and mortality after allogeneic hematopoietic stem cell transplantation (HSCT) [1]. We and others have proposed that measuring CMV-specific cellular immunity might help identify patients at risk for CMV infection and reactivation following HSCT [2,3,4,5,6,7,8,9]. Existing CMV-specific immune monitoring assays are based on the quantification of the number and/or functionality of CMV-specific immune cells using various detection technologies: flow cytometry (following intracellular or tetramer staining), enzyme-linked immunosorbent assay (ELISA) or enzyme-linked immunospot (ELISpot) [10,11,12]. Two standardized CMV-specific IFN-γ ELISpot assays, based on the in vitro stimulation of peripheral blood mononuclear cells (PBMC) with pp65 and IE-1 peptides (T-SPOT^®^.CMV) or T-activated^®^ proteins (T-Track^®^ CMV), recently demonstrated their suitability to measure CMV-specific cellular immunity in clinical settings [4,5,8,9,13,14,15,16,17,18].

To act as stimulant, exogenous proteins must be internalized and processed by antigen-presenting cells (APC; mainly dendritic cells and monocytes/macrophages). Internalized proteins are degraded into peptides within endosomes and associate with free major histocompatibility complex (MHC) class II and MHC class I (cross-presentation) molecules in the late endosomal compartment. Peptide-loaded MHC class I and II molecules are transported to the plasma membrane and presented to CD8^+^ and CD4^+^ T cells, respectively [19,20]. Epitope-specific T cells are then activated, leading to IFN-γ secretion [12]. Exogenous peptides are also taken up by APC and intracellularly loaded to *de novo*-synthesized MHC molecules before being transported to the cell surface and presented to T cells. In addition, exogenous peptides can be directly loaded to free MHC class I molecules present at the plasma membrane. High-affinity peptides can even compete with and replace peptides in preloaded MHC class I molecules at the cell surface. On the other hand, direct loading of peptides onto MHC class II molecules at the cell surface is unlikely to occur, due to the high stability of peptide-bound MHC class II complexes [19,21,22].

One might anticipate that these differences in antigen presentation may have an impact on the stimulating capability of CMV proteins and peptides, and thus on CMV-specific IFN-γ ELISpot test results. A recent study using intracellular cytokine staining and flow cytometry directly compared the ability of T-activated^®^ proteins and corresponding overlapping peptide pools to activate CD4^+^ and CD8^+^ T cells in healthy donors. Interestingly, T-activated^®^ proteins and peptides induced comparable CD4^+^ T-cell responses but significantly distinct CD8^+^ T-cell responses [23]. The questions remain as to how these differences in T-cell responses translate in a clinical setting, notably in immunocompromised patients with potentially impaired antigen processing, and how they influence CMV-specific IFN-γ ELISpot test results.

The present study directly compared the ability of CMV pp65 and IE-1 proteins and respective 15-mer overlapping peptides to induce the in vitro response of isolated PBMC of 16 HSCT recipients using a standardized CMV-specific IFN-γ ELISpot assay. The ELISpot results were interpreted in the context of the patient’s overall clinical picture and of total CD8^+^ T cell count.

Paired qualitative protein- and peptide-based test results were overall 74.4% concordant. However, one patient with early and sustained CMV reactivation and high CD8^+^ counts showed successive negative ELISpot results in response to CMV proteins but a high and sustained response to IE-1 peptides. These observations suggest that in some cases the response to exogenous peptides may lead to artificial in vitro T-cell responses, while the response to exogenous proteins, via their uptake and processing by antigen-presenting cells, more closely reflects the physiological response to CMV infection, especially in patients under intense immunosuppressive therapy.

## 2. Materials and Methods

### 2.1. Study Design and Participants

This study was performed as part of the registered and reported AlloProtectCMV study (clinicaltrials.gov identifier: NCT02156479; [4]). Spare peripheral blood mononuclear cells (PBMC) were used to conduct additional T-cell-based assays for research purposes only, in accordance with the study protocol. Study design, inclusion and exclusion criteria, and treatment regimen have been described elsewhere [4]. This study was approved by the respective ethics committees (DIMDI’s registration number 00008544; University of Regensburg’s approval number 13-122-0282, 28 February 2014) and all subjects gave written informed consent in accordance with the Declaration of Helsinki, as previously reported [4]. This investigation aimed to compare the response of isolated PBMC to cytomegalovirus (CMV) antigens used as either proteins or peptides in an IFN-γ ELISpot assay. CMV-specific cellular immunity was evaluated in the context of CD8^+^ T cell count, as other immune parameter, and of the overall clinical picture of individual patients, including CMV viral load, occurrence of CMV reactivation, CMV disease and graft-versus-host disease (GvHD). Sixteen intermediate- and high-risk (D+/R+, D+/R−, D−/R+) allogeneic HSCT recipients were included in this study. One to five IFN-γ ELISpot assays were conducted per patient and 49 ELISpot tests were performed in total.

### 2.2. Viral Load Measurement

CMV load was measured by quantitative PCR, as described [4]. CMV reactivation was defined as CMV viral load requiring antiviral treatment according to center-specific guidelines and/or physician’s decision [4].

### 2.3. IFN-γ ELISpot Assays

Blood collection, PBMC isolation and T-Track^®^ CMV assays (Lophius Biosciences GmbH, Regensburg, Germany) were performed and interpreted as previously described [4]. Spot-forming cells (SFC) were normalized to 200,000 PBMC and evaluated on the basis of square-root transformation (sqrt-SFC). Briefly, a test was considered positive if the mean of four replicate sqrt-SFC for 200,000 PBMC (SRM) resulting from pp65 and/or IE-1 stimulation was ≥sqrt(10) and if the difference of the mean of sqrt-SFC of the stimulated condition to that of the unstimulated condition (SRM[stimulated]—SRM[unstimulated]) was ≥0.742. SRM SFC values from unstimulated conditions were subtracted from those of the antigen-stimulated conditions. For a better display of pp65- and IE-1-specific SFC levels, SRM SFC values are presented as squared (SRM^2^) SFC values (i.e., as “spot count-equivalent”), and in a log10 scale. SRM^2^ SFC values are depicted as scattered plots showing median values (horizontal line), as before [4]. Peptide-based ELISpot assays were interpreted using the same approach and rules as the protein-based T-Track^®^ CMV assays.

### 2.4. CMV Antigens

T-activated^®^ CMV proteins pp65 (amino acids 366–546, hCMV strain AD169) and full length IE-1 (amino acids 1–491, hCMV Towne strain) are part of the T-Track^®^ CMV kit, as already described [12]. Peptide pools covering the respective pp65 (366–546) and IE-1 (1–491) regions were ordered from peptides&elephants (Hennigsdorf, Germany) as 15-mers overlapping by 11 amino acids. Accordingly, the respective peptide pools included 44 (pp65) and 120 (IE-1) peptides. Comparable pp65- and IE-1-specific libraries of 15-mer peptides with an 11 amino acid overlap have been widely described in the literature to study the response of CMV-specific CD4^+^ and CD8^+^ T cells [24,25,26]. Peptide pools were reconstituted in DMSO and used at a final concentration of 1 µg/mL/peptide (pp65) and 10 µg/mL/peptide (IE-1) per stimulation (19 h at 37 °C), based on titration experiments (Figure S1). The final DMSO concentration in stimulation experiments did not exceed 0.2%, and did not induce a non-specific response (Figure S1). Moreover, an independent experiment demonstrated a viability of PBMC after 19 h stimulation at 37 °C > 93% and no DMSO-associated toxicity (not shown), in agreement with published data [27]. Preliminary flow cytometry experiments indicated that pp65 and IE-1 proteins and peptides both activated IFN-γ-producing CD4^+^ and CD8^+^ T cells (not shown), in line with recently published results [23].

### 2.5. Total CD8^+^ T Cell Count Determination

Total CD8^+^ cells were enumerated by flow cytometry from the same PBMC, and absolute cell counts were calculated using the peripheral blood absolute lymphocyte count determined at the same visit, as previously described [4].

### 2.6. Statistical Analysis

Differences in pp65- and IE-1-specific SFC distributions between ELISpot tests performed using either T-activated^®^ proteins or overlapping peptides were tested using the Mann-Whitney U (MWU) test. Two-sided *p*-values < 0.05 were considered statistically significant. Due to (i) the existence of multiple measurements per patient (in 9/15 patients), (ii) the heterogeneity of the number of measurements per patient (one ELISpot assay in six patients and two to five ELISpot assays in nine patients), and (iii) the low case number (15 patients included in the final analysis), no statistical tests were applied to compare test agreement in response to proteins vs. peptides. This study aimed at comparing the response to proteins and peptides to identify and characterize potential discordant test results in the context of the overall clinical picture.

## 3. Results

### 3.1. Patient Characteristics

**Figure 1:**
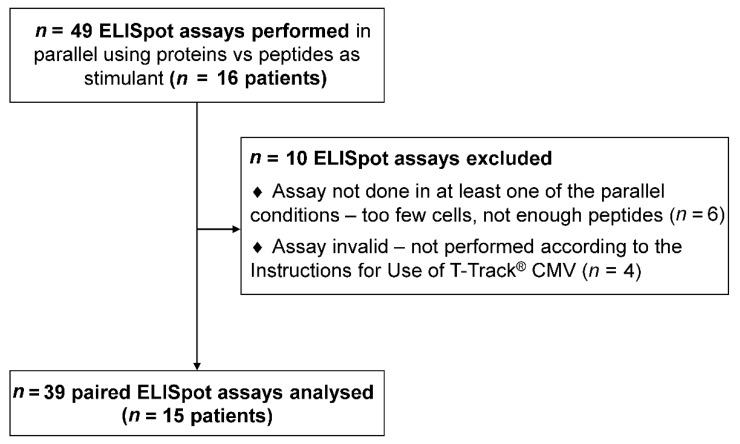
Study flow diagram. Sixteen patients were included in this study. One to five enzyme-linked immunospot (ELISpot) assays were conducted per patient, using pp65 and IE-1 proteins and peptide pools in parallel for PBMC stimulation. A total of 49 ELISpot assays were performed. Ten ELISpot assays were excluded from the analysis due to missing data in at least one of the stimulating conditions. Thirty-nine valid paired ELISpot measurements (15 patients) were included in the final analysis (Table 1).

Sixteen HSCT patients were included in this study. A total of 49 ELISpot assays were performed, applying pp65 and IE-1 proteins and peptide pools in parallel for PBMC stimulation. Ten ELISpot assays were excluded from the analysis due to missing data in at least one of the stimulating conditions (Figure 1).

Thirty-nine valid paired ELISpot measurements in 15 patients (one to five tests per patient) were included in the final analysis. Patient characteristics are shown in Table 1. All collected parameters are shown in Table S1.

### 3.2. Measurement of CMV-Specific Cellular Immunity in Response to CMV Proteins and Peptides

CMV-specific cell-mediated immunity was evaluated using a standardized IFN-γ ELISpot-based assay (T-Track^®^ CMV), which employs urea-formulated (T-activated^®^) pp65 and IE-1 proteins as stimulant [4,12,13,14,22]. PBMC were stimulated in parallel with pp65 and IE-1 15-mer peptides covering the same amino acid regions, respectively, and analyzed using the same ELISpot assay procedure and reagents.

**Figure 2:**
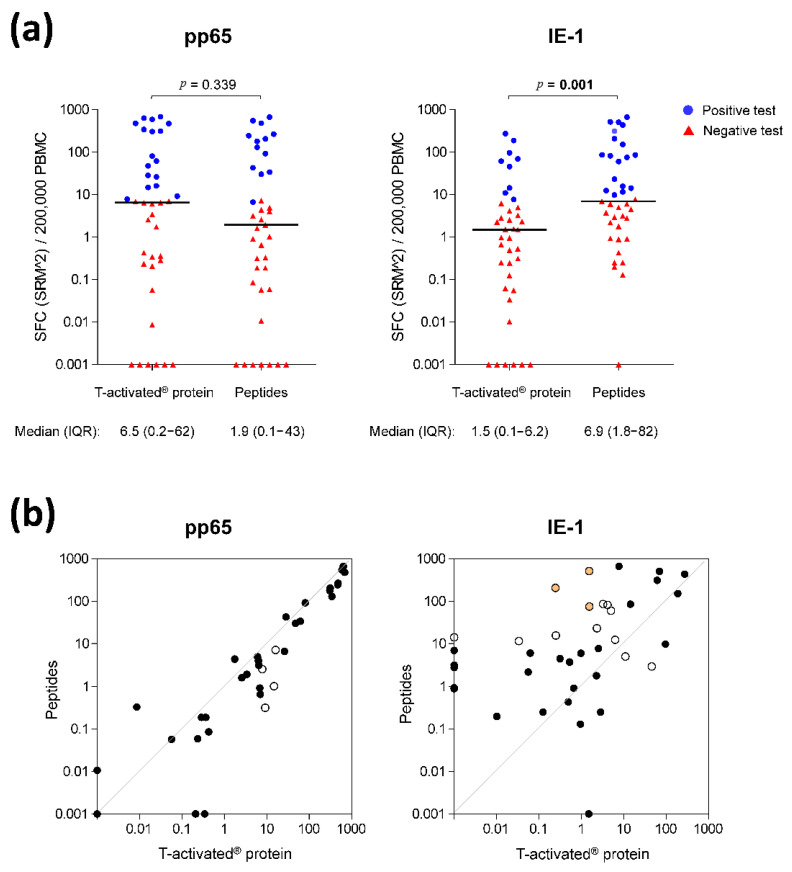
CMV-specific cell-mediated immunity in response to pp65 and IE-1 T-activated^®^ proteins and respective overlapping peptides. (**a**) Quantitative IFN-γ ELISpot results in response to CMV T-activated^®^ proteins pp65 and IE-1 and to the respective overlapping peptides (normalized to 200,000 PBMC) were evaluated on the basis of the mean of square-root-transformed (SRM) spot-forming cells (SFC), as previously described [4]. Differences in SFC distribution in response to proteins and peptides were evaluated using a Mann-Whitney-U test. Statistically significant *p*-values are shown in bold above each graph. For the sake of simplicity, scatter plots are depicted as squared SRM values (SRM^2^). Median and interquartile range (IQR) of SRM^2^ SFC are shown under each graph. Due to the log scale representation, values of zero SRM^2^ were replaced by 0.001 (*y*-axis), meaning that baseline values shown at *y* = 0.001 are actually equal to zero. Red triangles and blue dots depict negative and positive tests, respectively, defined according to the rules described in the Methods section and previously reported [4]. (**b**) Paired quantitative IFN-γ ELISpot results in response to T-activated^®^ proteins versus peptides. The antigen-specific ELISpot results shown in panel (a) are represented as paired data. Black circles depict overall concordance (positive and negative concordance; Table 2) and white circles depict discordant results (Figure 3). Orange-filled circles indicate the highly-discordant test results observed in response to IE-1 proteins and peptides in patient no. 12 (Figure 4).

The overall spot-forming cells (SFC) distribution was comparable in response to pp65 proteins and peptides (MWU-test *p* = 0.339), while the response to IE-1 was significantly higher in response to peptides (MWU-test *p* = 0.001) (Figure 2a). The analysis of the paired quantitative results showed the same trend (Figure 2b).

The analysis of the paired qualitative (positive/negative) test results revealed that 35/39 (89.7%) and 26/39 (66.7%) tests were concordant in response to pp65 and IE-1, respectively (Figure 2b, black circles; Table 2). The proportion of concordant overall ELISpot test results (accounting for both pp65 and IE-1 response, according to the manufacturer’s instructions) was 74.4% (29/39; Table 2).

**Figure 3:**
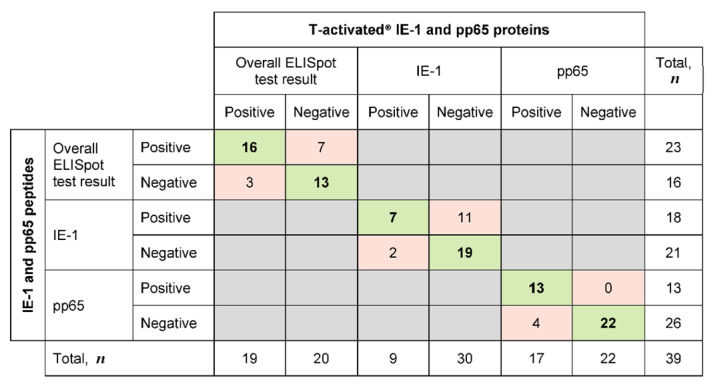
Concordance and discordance of qualitative ELISpot test results following stimulation of PBMC with CMV proteins or peptides (*n* = 39). Qualitative IFN-γ ELISpot results in response to CMV T-activated^®^ proteins pp65 and IE-1 and to the respective overlapping peptides (normalized to 200,000 PBMC) were evaluated on the basis of the mean of square-root-transformed (SRM) spot-forming cells (SFC), as previously described [4]. Overall ELISpot test results were interpreted as positive when at least one of the IE-1- and/or pp65-specific response was positive, and as negative when both IE-1 and pp65-specific responses were negative. The number of concordant tests (either positive or negative with both tests) is highlighted in green, while the number of discordant tests (positive/negative) between protein- and peptide-based ELISpot assays is highlighted in red.

A detailed evaluation of concordant and discordant positive and negative test results is shown in Figure 3 (green and red fields, respectively). The evaluation of the discordant qualitative test results revealed four and 13 discordant pp65- and IE-1-specific test results, respectively (Figure 2b, open circles; Figure 3, red fields; Table S1, greyed fields). The four discordant pp65 test results (positive in response to proteins and negative in response to peptides) showed low spot counts (SFC [SRM^2^]/200,000 PBMC ranging from 0.31 to 16.1; Table S1, patients no. 10, 11 and 15), hence close to the limit of quantitation (LoQ) of the ELISpot assay. These four tests were therefore not considered as truly discordant. Six out the 13 discordant IE-1 test results showed a similar pattern of low spot counts near the LoQ (SFC [SRM^2^]/200,000 PBMC ranging from 0.00 to 23.2; Table S1, patients no. 2, 3, 8, 11 and 14), therefore not truly discordant either. Out of the remaining seven discordant IE-1 tests, four were positive for both pp65 proteins and peptides (Table S1, patients no. 5, 8 and 13), thus resulting in an overall positive (concordant) ELISpot test. In these four cases, the IE-1 discordance had therefore no impact on the final test result (overall positive and concordant between proteins and peptides). The remaining three discordant tests belonged to one patient (no. 12). The response to IE-1 protein was negative (SFC [SRM^2^]/200,000 PBMC ranging from 0.25 to 1.55; Table S1) while that to IE-1 peptides was positive and showed high spot counts (SFC [SRM^2^]/200,000 PBMC ranging from 75.3 to 514.4; Table S1). The response to pp65 protein and peptides was negative in all cases (SFC [SRM^2^]/200,000 PBMC ranging from 0.00 to 0.42; Table S1). These truly discordant test results in a single patient were investigated in more detail.

### 3.3. Monitoring of Immune Parameters and Clinical Complications in Patient No. 12

**Figure 4:**
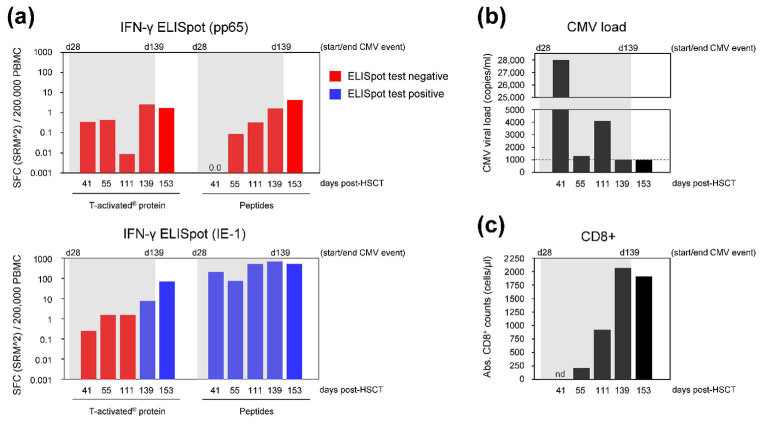
Immune parameters and CMV viral load of HSCT recipient no. 12. Blood of patient no. 12 was withdrawn at day 41, 55, 111, 139 and 153 post-HSCT. (**a**) PBMC were isolated and analyzed by IFN-γ ELISpot using CMV T-activated^®^ proteins or respective overlapping peptides as stimulant. Negative and positive ELISpot test results are represented in red and blue, respectively. ELISpot results were negative at all visits upon stimulation with pp65 T-activated^®^ protein and peptides. Similarly, the response to T-activated^®^ IE-1 protein was low during CMV reactivation (between 0.25 and 8 SFC [SRM^2^]/200,000 PBMC), progressively increasing overtime (up to 69 SFC [SRM^2^]/200,000 PBMC at day 153). In contrast, IE-1-specific response to peptides was high at all investigated visits (75 to 667 SFC [SRM^2^]/200,000 PBMC). (**b**) CMV viral load (VL), expressed in copies/mL blood, was measured in parallel. CMV VL was above the center-specific threshold for pre-emptive antiviral therapy (dashed line) at day 41 to 111 and returned to the institutional threshold at day 139 and 153. (**c**) Remaining PBMC were used for the determination of total CD8^+^ T cells by flow cytometry (expressed as total CD8^+^ T cells/µL blood). Flow cytometry was not performed at day 41 (nd, not determined) due to a lack of PBMC. Absolute CD8^+^ counts were high, ranging from 212 to 2065 cells/µL blood at all investigated visits (day 55 to 153). In (**a**–**c**), grey shading represents the lengthy CMV event (CMV reactivation requiring antiviral treatment), from day 28 to 139 post-HSCT. Of note, treatment was temporarily interrupted between day 61 and 98 due to drug toxicity. CMV disease was documented at day 28 post-HSCT (i.e., before visit 1/day 41). Acute GvHD (grade I) was also documented before day 41, while no GvHD was documented thereafter (Table S1). Of note, GvHD prophylaxis using the calcineurin inhibitor cyclosporin was discontinued at day 132 post-transplantation.

The five available IFN-γ ELISpot measurements for patient no. 12 (day 41 to 153 post HSCT) were evaluated in parallel to the respective total CD8^+^ T cell count, as well as to the respective CMV viral load and other clinical complications (Figure 4 and Table S1).

Patient no. 12 belonged to the high-risk CMV serology group (D−/R+). CMV disease (encephalitis) was documented at day 28 post HSCT. The patient presented high, sustained CMV DNAemia ranging from 1300 to 28,000 copies/mL blood between day 41 and 111, which dropped below the institutional threshold of 1000 copies/mL at day 139 and 153 (Figure 4b; Table S1). Antiviral therapy was initiated at day 28 and discontinued at day 139 after transplantation (with a 37-day interruption between day 61 and 98 due to drug-induced leukopenia). Acute GvHD (grade I) was documented before visit 1 (day 20 to 33 post HSCT) and was treated topically with corticosteroids in addition to the continued cyclosporin prophylaxis. No GvHD was documented thereafter (Table S1) and cyclosporin prophylaxis was discontinued at day 132 after transplantation. Absolute CD8^+^ T cell counts were available at day 55 to 153. CD8^+^ T cell counts drastically increased over time, ranging from 212 to 2065 cells/µL blood (Figure 4c and Table S1).

The IFN-γ ELISpot response to pp65 protein and respective peptides was negative at all investigated visits (Figure 4a). The response to T-activated^®^ IE-1 protein was negative at day 41, 55 and 111 post HSCT, turning positive and increasing afterward, and reaching 69 SFC (SRM^2^)/200,000 PBMC at day 153 (Figure 4a and Table S1). In contrast, IE-1-specific response to peptides was positive and high at all investigated visits, ranging from 75 to 667 SFC (SRM^2^)/200,000 PBMC (Figure 4a and Table S1). Accordingly, the overall T-Track^®^ CMV test in response to T-activated^®^ proteins was negative at day 41, 55 and 111, during the treated CMV reactivation, and positive at day 139 and 153, after the end of treatment for CMV reactivation. In comparison, the overall ELISpot test in response to peptides was positive at all visits, due to the high spot counts induced by IE-1 peptide stimulation.

## 4. Discussion

This study compared the response of PBMC to CMV antigens in a standardized IFN-γ ELISpot assay, using either proteins or respective peptides as stimulant. Based on 39 ELISpot assays performed in parallel (including 15 HSCT recipients), the overall test concordance (accounting for both pp65 and IE-1 response) was 74.4%. Test concordance was stronger in response to pp65 (89.7%) than to IE-1 (66.7%). The lower concordance in response to IE-1 was due to the significantly higher response to peptides vs. proteins, resulting in a higher proportion of positive test results. The higher response to IE-1 peptides might reflect a difference in antigen presentation between proteins and peptides, possibly due to the direct loading of exogenous peptides to MHC class I molecules on the cell surface of APC [19,21,22]. That a higher T-cell response was observed using IE-1 but not pp65 peptides might be due to a higher affinity of IE-1 peptides to the peptide-binding groove of MHC class I molecules [21], although this proposition remains to be demonstrated.

Discordant qualitative (positive/negative) test results in response to proteins vs. peptides were mainly those with very low spot counts and thus close to the LoQ, with one exception. One patient showed repetitively negative T-Track^®^ CMV tests (in response to T-activated^®^ pp65 and IE-1 proteins) but positive tests with high SFC counts in response to IE-1 (but not pp65) peptides. This patient was characterized by the occurrence of aGvHD and CMV disease early after transplantation, high and sustained CMV DNAemia despite antiviral treatment, and elevated and increasing total CD8^+^ counts.

The successively negative T-Track^®^ CMV tests correlated with the high sustained CMV viral load, in line with the expectation that compromised CMV-specific cellular immunity is unable to contain CMV reactivation. Following discontinuation of GvHD prophylaxis at day 132, the T-Track^®^ CMV test turned positive (with low spot counts) at day 139, as viral load went down and antiviral treatment was stopped. T-Track^®^ CMV was then confirmed positive at day 153, with increasing spot counts (69 SFC [SRM^2^]/200,000 PBMC), indicating progressive CMV-specific immune reconstitution. This chronology of events also suggests a potential functional interference of cyclosporin with the response to CMV proteins. Altogether, T-Track^®^ CMV test results were in accordance with the patient’s clinical picture in terms of risk stratification for CMV complications.

In contrast, the high response to IE-1 peptides at all investigated visits was unexpected. This elevated response might reflect the frequency of reactive CD8^+^ T cells upon presentation by exogenous peptides directly loaded to MHC class I molecules at the cell surface, rather than a physiological immune response, as reported for other viral peptide antigens [28,29,30]. This proposition is in agreement with the recent demonstration that the stimulation of PBMC of healthy donors with pp65 and IE-1 overlapping peptides results in a stronger response of CD8^+^ (but not CD4^+^) T cells, compared to the stimulation with the respective T-activated^®^ proteins [23]. In line with these observations, patient no. 12 presented increased total CD8^+^ T cell counts. Whether high CD8^+^ T cell counts reflected equally elevated levels of CMV-specific CD8^+^ T cells in this patient is not known, although absolute CD8^+^ T cell counts usually correlate well with CMV-specific CD8^+^ T cell levels in peripheral blood [11,31,32,33].

The lack of PBMC responsiveness to pp65 and IE-1 proteins in patient no. 12 cannot be explained by the patient’s HLA genotype, since it included several common alleles (e.g., HLA-A*02:01 and HLA-B*07:02) known to bind immunodominant pp65 and IE-1 epitopes [34,35,36]. Instead, the lack of PBMC responsiveness to pp65 and IE-1 proteins might indicate impaired antigen processing and presentation by APC [22]. Indeed, immunosuppressive agents such as corticosteroids or calcineurin inhibitors are known to interfere with dendritic cells’ function [37,38,39], and patient no. 12 was treated topically with corticosteroids in addition to GvHD prophylaxis with a calcineurin inhibitor at the time CMV disease was diagnosed. Besides, following discontinuation of cyclosporin prophylaxis at day 132, the response to IE-1 protein became positive at day 139 and the number of IE-1-reactive cells in ELISpot further increased at day 153.

Altogether, the overall clinical picture of this patient and the measured immune parameters suggest that the elevated sustained response to IE-1 peptides in the IFN-γ ELISpot assay indicate the presence of high levels of CMV-specific CD8^+^ T cells in peripheral blood, rather than their functionality. In contrast, the lack of response to T-activated^®^ pp65 and IE-1 proteins in the IFN-γ ELISpot assay apparently reflects the lack of in vivo protection, likely due to impaired antigen uptake, processing and presentation. This proposition is further supported by reports of impaired in vivo protection against CMV in HSCT patients with GvHD under intense immunosuppressive therapy, despite the detection of high levels of CMV-specific CD8^+^ T cells or of in vitro IFN-γ CD8^+^ response to CMV-specific peptides [40,41].

## 5. Conclusions

This study demonstrated comparable IFN-γ ELISpot results in response to pp65 and IE-1 proteins and peptides in most cases (14 out of 15 patients). Yet, some patients, especially those under strong immunosuppressive therapy, might show unreliable in vitro T-cell responses to peptides. In vitro T-cell responses to proteins seem to mimic more closely the natural response to CMV infection via the antigen uptake, processing and presentation pathway, hence allowing more accurate and reliable risk stratification of patients. This study further demonstrates the suitability of the standardized IFN-γ ELISpot assay T-Track^®^ CMV to evaluate CMV-specific cellular immunity in immunocompromised patients, and supports its implementation in clinical routine for an improved risk stratification and management of patients.

## Figures and Tables

**Table 1:** Patient characteristics.

Study Population, *n* (%)	15 (100%)
Gender, *n* (%)	
Male	8 (53.3%)
Female	7 (46.7%)
Age in years, median (range)	57 (29–70)
Underlying disease, *n* (%)	
Acute myeloid leukemia	10 (66.7%)
Acute lymphoid leukemia	2 (13.3%)
Non-Hodgkin’s lymphoma	2 (13.3%)
Severe aplastic anemia	1 (6.7%)
Donor (D)/Recipient (R) CMV serostatus, *n* (%)	
D+/R+	4 (26.7%)
D+/R−	1 (6.6%)
D−/R+	10 (66.7%)
Stem cell source, *n* (%)	
Bone marrow	3 (20.0%)
Peripheral blood	12 (80.0%)
Donor source, *n* (%)	
Matched sibling	4 (26.7%)
Matched unrelated donor	9 (60.0%)
Mismatched unrelated donor	2 (13.3%)
Conditioning regimen, *n* (%)	
Non-myeloablative	3 (20.0%)
Myeloablative, standard (MAC)	9 (60.0%)
Myeloablative, toxicity-reduced (RIC)	3 (20.0%)
At least one treatment-requiring CMV reactivation, *n* (%)	10 (66.7%)
CMV disease, *n* (%)	1 (6.7%)
Graft-versus-host disease (GvHD), *n* (%)	6 (40.0%)
Death, *n* (%)	2 (13.3%)

**Table 2:** Concordance of qualitative ELISpot test results following stimulation of PBMC with CMV proteins or peptides (*n* = 39).

Results	Positive Concordance	Negative Concordance	Overall Concordance	
	*n*	*n*	*n*	%
Overall ELISpot test Result *^a^*	16	13	29	74.4% (29/39)
IE-1	7	19	26	66.7% (26/39)
pp65	13	22	35	89.7% (35/39)

*^a^* Test is positive when at least one of the IE-1- and/or pp65-specific response is positive; test is negative when both IE-1- and pp65-specific responses are negative.

## Data Availability

The data presented in this study are available within the manuscript and Supplementary Material (Table S1).

## Supplementary Materials

The following are available online at https://www.mdpi.com/2075-4418/11/2/312/s1, Table S1: Individual patients’ characteristics, and measured immune and CMV viral load parameters. Figure S1. Titration of pp65 and IE-1 peptide pools.
