# Supplementary material for: Comparison of Cytomegalovirus-Specific Immune Cell Response to Proteins versus Peptides Using an IFN-γ ELISpot Assay after Hematopoietic Stem Cell Transplantation
Source: Diagnostics (Basel). 2021 Feb 15;11(2):312. doi: 10.3390/diagnostics11020312 (PMC7919014; doi:10.3390/diagnostics11020312)
Supplement: Supplementary file 1 [file diagnostics-11-00312-s001.zip › diagnostics-1060132-supplementary/Figure S1.pdf]

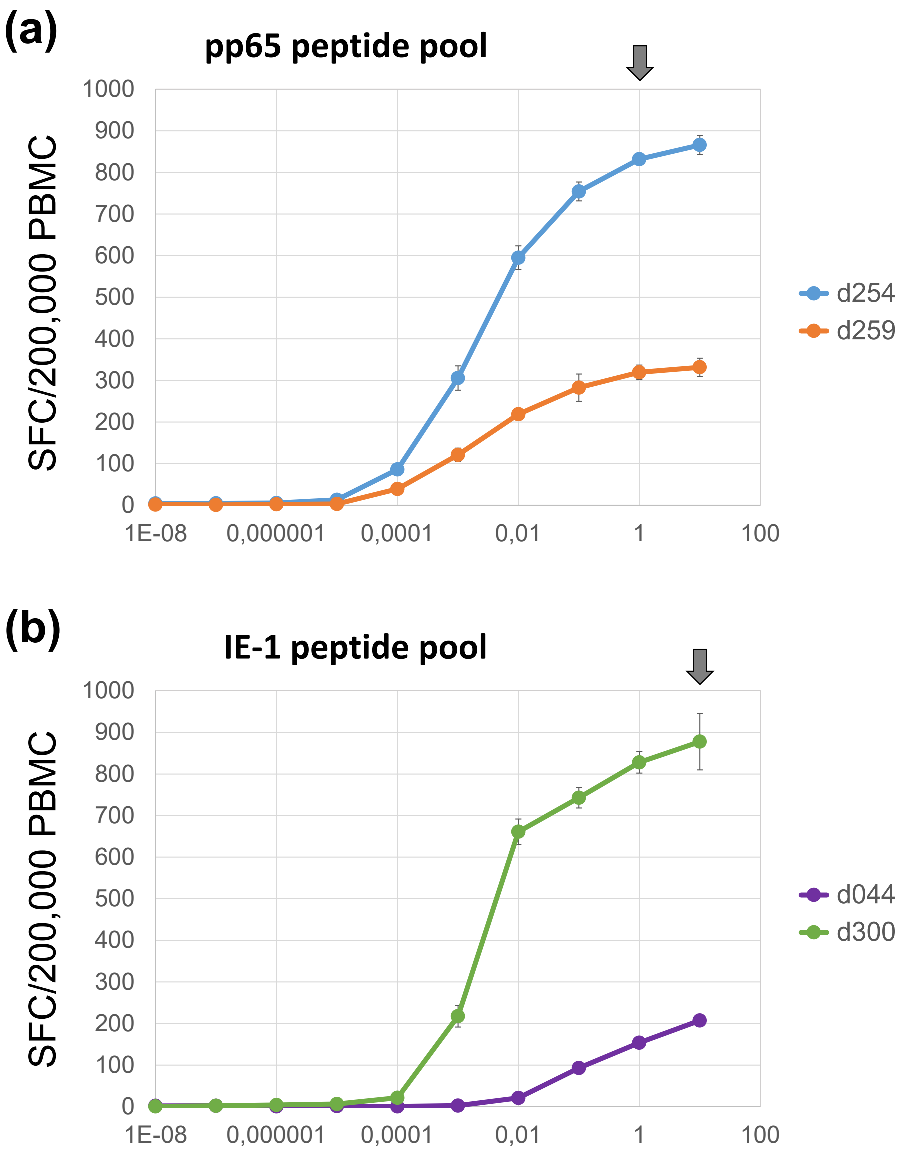

Figure S1. Titration of pp65 and IE-1 peptide pools. Isolated PBMC of two CMV-seropositive healthy donors each were stimulated in quadruplicates for 19h at 37°C with the indicated concentrations of pp65 (**a**) or IE-1 (**b**) peptide pools, and IFN-γ ELISpot assays were performed as described in Materials and Methods. Mean (±SD) spot forming cells (SFC) per 200,000 PBMC are depicted as a function of the concentration of peptides used for the stimulation. The selected concentration used in following experiments is indicated by the grey arrow above each graph: 1 µg/mL for pp65 (in 0.01% DMSO final) and 10 µg/mL for IE-1 (in 0.2% DMSO final). Given the higher concentration of DMSO employed per stimulation with the IE-1 peptide pool in (b), a DMSO control (0.2% in medium) was tested in parallel and yielded mean SFC/200,000 PBMC of 2.0 (donor d044) and 1.5 (donor d300), equivalent to background level.
