# Supplementary material for: Comparison of Cytomegalovirus-Specific Immune Cell Response to Proteins versus Peptides Using an IFN-γ ELISpot Assay after Hematopoietic Stem Cell Transplantation
Source: Diagnostics (Basel). 2021 Feb 15;11(2):312. doi: 10.3390/diagnostics11020312 (PMC7919014; doi:10.3390/diagnostics11020312)
Supplement: Supplementary file 1 [file diagnostics-11-00312-s001.zip › diagnostics-1060132-supplementary/Table S1.pdf]

Supplemental Table 1. Overview of patients' characteristics and measured parameters

Patient no.	Age	Gender	Under-lying disease	CMV serology	Donor source	Stem cell source	Condition-ing regimen	Visit no.	Day after HSCT	T-activated® proteins: IE-1 SFC [SRM^2] / 200,000 PBMC	T-activated® proteins: IE-1 Qualitative test result	T-activated® proteins: pp65 SFC [SRM^2] / 200,000 PBMC	T-activated® proteins: pp65 Qualitative test result	T-activated® proteins: Overall test Qualitative test result	Peptides: IE-1 SFC [SRM^2] / 200,000 PBMC	Peptides: IE-1 Qualitative test result	Peptides: pp65 SFC [SRM^2] / 200,000 PBMC	Peptides: pp65 Qualitative test result	Peptides: Overall test Qualitative test result	CD8+ (cells/µl)	VL (copies/ml) [bold: VL>threshold]	Number of documented CMV reactivations [*: additional event before visit1/d45]	Documented CMV disease	Start / End day of CMV reactivation (rel. to day of Tx)	Ongoing CMV reactivation	Any documented GvHD	Ongoing GvHD	Other AE
1	46	female	NHL	D+/R-	Matched unrelated	PBSC	MAC	3	76	0,06	Negative	47,55	Positive	Positive	2,19	Negative	30,34	Positive	Positive	41,70	0	0	No	N/A	N/A	Yes (before d45/v1 only: aGvHD, I)	N/A	
2	70	male	AML	D+/R+	Mismatched unrel.	PBSC	MAC	4	111	6,22	Negative	6,34	Negative	Negative	12,42	Positive	4,01	Negative	Positive	1053,40	0	0	No	N/A	N/A	No	N/A	
3	40	female	ALL	D-/R+	Matched sibling	PBSC	MAC	2	65	0,53	Negative	0,00	Negative	Negative	3,74	Negative	0,00	Negative	Negative	876,84	250	0	No	N/A	N/A	Yes (v4, v5)	No	
								4	104	0,31	Negative	0,00	Negative	Negative	4,51	Negative	0,00	Negative	Negative	845,98	250				N/A		Yes (aGvHD, I)	
								5	119	0,03	Negative	0,00	Negative	Negative	11,59	Positive	0,01	Negative	Positive	732,24	250				N/A		Yes (aGvHD, I)	
4	43	female	AML	D-/R+	Matched sibling	BM	MAC	1	44	0,12	Negative	0,00	Negative	Negative	0,25	Negative	0,00	Negative	Negative	missing	0	0	No	N/A	N/A	Yes (before d45/v1 only: aGvHD, II)	N/A	
								4	106	2,83	Negative	0,00	Negative	Negative	0,25	Negative	0,00	Negative	Negative	missing	0				N/A		N/A	
5	61	male	AML	D-/R+	Matched sibling	PBSC	MAC	1	52	14,37	Positive	681,44	Positive	Positive	85,50	Positive	480,99	Positive	Positive	589,64	250	0	No	N/A	N/A	Yes (before d45/v1: aGvHD, I; v1, v6)	Yes (aGvHD, I)	
								2	62	4,15	Negative	633,40	Positive	Positive	81,70	Positive	667,23	Positive	Positive	1419,37	250	0			N/A		No	
								3	80	3,24	Negative	588,10	Positive	Positive	86,39	Positive	551,98	Positive	Positive	1464,07	250	0			N/A		No	
								6	199	2,53	Negative	304,67	Positive	Positive	7,68	Negative	178,86	Positive	Positive	907,73	250	0			N/A		Yes (cGvHD, m)	
6	60	male	NHL	D+/R+	Matched unrelated	PBSC	MAC	1	45	187,86	Positive	476,54	Positive	Positive	151,32	Positive	266,96	Positive	Positive	909,34	250	1	No	41 / 61	Yes	No	N/A	
7	59	male	AML	D+/R+	Matched unrelated	PBSC	Non-MAC	4	76	272,02	Positive	468,12	Positive	Positive	435,12	Positive	245,49	Positive	Positive	missing	50	1	No	27 / 48	No	No	N/A	Death (d208)
8	59	female	AML	D-/R+	Matched unrelated	PBSC	Non-MAC	1	43	10,90	Positive	61,74	Positive	Positive	5,01	Negative	33,70	Positive	Positive	1460,66	**1700**	1*	No	48 / 82	No	No	N/A	
								3	69	0,01	Negative	6,03	Negative	Negative	0,20	Negative	4,92	Negative	Negative	743,78	150	1*			Yes		N/A	
								5	98	45,53	Positive	28,26	Positive	Positive	2,97	Negative	42,51	Positive	Positive	753,21	50	1*			No		N/A	
								6	131	96,22	Positive	80,66	Positive	Positive	9,78	Positive	92,18	Positive	Positive	missing	50	1*			No		N/A	
9	64	female	AML	D+/R+	Matched unrelated	PBSC	MAC	2	51	61,00	Positive	311,44	Positive	Positive	312,36	Positive	204,51	Positive	Positive	missing	260	1	No	51 / 71	Yes	No	N/A	Death (d188)
10	29	male	ALL	D-/R+	Matched unrelated	PBSC	MAC	2	51	0,67	Negative	7,00	Negative	Negative	0,91	Negative	0,65	Negative	Negative	877,66	**60000**	1	No	58 / 79	No	No	N/A	
								3	62	0,49	Negative	3,39	Negative	Negative	0,43	Negative	1,93	Negative	Negative	1092,96	300	1			Yes		N/A	
								4	79	0,95	Negative	7,84	Positive	Positive	0,13	Negative	2,57	Negative	Negative	1189,97	250	1			Yes		N/A	
								6	93	2,27	Negative	26,04	Positive	Positive	1,80	Negative	6,60	Positive	Positive	missing	250	1			No		N/A	
11	62	male	AA	D-/R+	Matched unrelated	BM	MAC	1	42	0,00	Negative	0,36	Negative	Negative	2,79	Negative	0,19	Negative	Negative	61,94	**2700**	1*	No	44 / 76	No	No	N/A	
								2	55	2,33	Negative	16,09	Positive	Positive	23,21	Positive	7,10	Negative	Positive	137,47	**6500**	1*			Yes		N/A	
								3	76	0,00	Negative	0,24	Negative	Negative	3,20	Negative	0,06	Negative	Negative	missing	150	1*			Yes		N/A	
								4	80	0,00	Negative	0,06	Negative	Negative	6,93	Negative	0,06	Negative	Negative	170,77	missing	1*			No		N/A	
								6	104	0,98	Negative	14,78	Positive	Positive	5,97	Negative	1,02	Negative	Negative	111,34	150	1*			No		N/A	
12	53	female	AML	D-/R+	Mismatched unrel.	PBSC	RIC	1	41	0,25	Negative	0,34	Negative	Negative	206,38	Positive	0,00	Negative	Positive	missing	**28000**	1	Yes (d28)	28 / 139	Yes	Yes (before d45/v1 only: aGvHD, I)	N/A	
								2	55	1,55	Negative	0,42	Negative	Negative	75,28	Positive	0,09	Negative	Positive	212,03	**1300**	1			Yes		N/A	
								5	111	1,53	Negative	0,01	Negative	Negative	514,43	Positive	0,33	Negative	Positive	922,78	**4100**	1			Yes		N/A	
								6	139	7,66	Positive	2,58	Negative	Positive	666,74	Positive	1,61	Negative	Positive	2065,11	**1000**	1			Yes		N/A	
								7	153	69,41	Positive	1,75	Negative	Positive	504,02	Positive	4,40	Negative	Positive	1907,50	**1000**	1			No		N/A	
13	53	female	AML	D-/R+	Matched sibling	BM	Non-MAC	8	126	4,96	Negative	340,32	Positive	Positive	59,92	Positive	129,07	Positive	Positive	1242,96	0	2	No	26 / 56; 68 / 81	No	No	N/A	
14	57	male	AML	D-/R+	Matched unrelated	PBSC	RIC	3	76	0,00	Negative	0,00	Negative	Negative	0,89	Negative	0,00	Negative	Negative	9,94	300	2	No	35 / 76; 94 / 182	Yes	Yes (before d45/v1 only: aGvHD, III)	N/A	
								6	121	0,06	Negative	0,29	Negative	Negative	6,01	Negative	0,19	Negative	Negative	117,20	0	2			Yes		N/A	
								7	133	0,00	Negative	6,81	Negative	Negative	14,20	Positive	0,92	Negative	Positive	38,28	300	2			Yes		N/A	
								8	141	0,25	Negative	6,45	Negative	Negative	15,68	Positive	3,16	Negative	Positive	48,20	0	2			Yes		N/A	
15	57	male	AML	D-/R+	Matched unrelated	PBSC	RIC	1	40	1,49	Negative	0,21	Negative	Negative	0,00	Negative	0,00	Negative	Negative	14,54	**7600**	2*	No	42 / 67; 89 / 102	No	No	N/A	
								2	56	0,00	Negative	9,14	Positive	Positive	0,93	Negative	0,31	Negative	Negative	missing	**1200**	2*			Yes		N/A	

Grey fields indicate discordant qualitative test results in response to T-activated® proteins vs. Peptides. ELISpot assays were conducted from isolated PBMC according to the manufacturer's recommendations. Total CD8+ T cells were enumerated from the same PBMC preparation by flow cytometry, and expressed as absolute cell counts per microliter of blood.
ABBREVIATIONS: Underlying diseases: AA, aplastic anemia; ALL, acute lymphoid leukemia; AML, acute myeloid leukemia; NHL, non-hodgkin's lymphoma; CMV serology: D-, CMV-seronegative donor; D+, CMV-seropositive donor; R-, CMV-seronegative recipient; R+, CMV-seropositive recipient; Stem cell source: BM, bone marrow; PBSC, peripheral blood stem cells; Conditioning regimen: MAC, myeloablative conditioning (standard); RIC, reduced-intensity myeloablative conditioning; Non-MAC, non-myeloablative conditioning; ELISpot assays: IE-1, immediate-early 1 (CMV antigen); pp65, phosphoprotein 65 (CMV antigen); PBMC, peripheral blood mononuclear cells; SFC, spot-forming cells; SRM, mean of square-root-transformed replicate SFC values; SRM^2, squared SRM value (spot count equivalent); Cytomegalovirus (CMV): VL, viral load; Graft-versus-host disease (GvHD): aGvHD, acute GvHD; aGvHD I-III, aGvHD grade I-III; cGvHD, chronic GvHD; cGvHD m, cGvHD moderate; Other: AE, adverse events; d, day; d45/v1, day 45 after HSCT/visit 1; N/A, not applicable; v1-6, visits 1-6.
